# Development and validation of a predictive model for 30-day mortality in adult bacterial meningitis: a retrospective cohort study

**DOI:** 10.3389/fmed.2025.1692631

**Published:** 2025-11-20

**Authors:** Jun Zhou, Jicheng Xing, Xiangjun Cheng, Fei Jin, Yiling Huang

**Affiliations:** 1 Department of Laboratory Medicine, The First Affiliated Hospital With Nanjing Medical University, Nanjing, China; 2 Branch of National Clinical Research Center for Laboratory Medicine, Nanjing, China; 3 Institute of Laboratory Medicine, Jinling Hospital, Affiliated Hospital of Medical School, Nanjing University, Nanjing, China

**Keywords:** bacterial meningitis, mortality, prediction model, nomogram, risk factors

## Abstract

**Background:**

Bacterial meningitis continues to carry significant mortality despite advances in antimicrobial therapy. Early identification of high-risk patients remains challenging in clinical practice.

**Methods:**

We conducted a retrospective analysis of 277 adult patients with bacterial meningitis admitted between 2016 and 2024. Patients were randomly allocated to training (*n* = 194) and validation (*n* = 83) cohorts. Comprehensive clinical parameters, laboratory findings (including cerebrospinal fluid analysis), and microbiological data were collected. Least Absolute Shrinkage and Selection Operator (LASSO) regression and multivariable regression were used to construct a predictive nomogram. Model performance was assessed by area under the receiver operating characteristic curve (AUC), calibration plots, and decision curve analysis.

**Results:**

The overall 30-day mortality rate was 29.2% (81/277). In multivariate analysis, five independent predictors emerged: age [hazard ratio (HR) 1.04, 95% confidence interval (CI): 1.01–1.08], neurological complications (HR 2.31, 95% CI: 1.12–4.78), multidrug-resistant (MDR) infection (HR 3.15, 95% CI: 1.42–6.99), cerebrospinal fluid neutrophil percentage (HR 1.03, 95% CI: 1.01–1.05), and serum C-reactive protein (HR 1.12, 95% CI: 1.03–1.22). The nomogram demonstrated good discrimination with AUCs of 0.851 (95% CI: 0.793–0.909) in the training cohort and 0.814 (95% CI: 0.715–0.914) in validation. Decision curve analysis confirmed clinical utility across a wide probability threshold range.

**Conclusion:**

Our validated prediction model incorporating readily available clinical and laboratory parameters provides accurate risk stratification for adult bacterial meningitis patients. This tool may assist clinicians in identifying high-risk individuals who could benefit from more intensive monitoring and treatment strategies.

## Introduction

Bacterial meningitis, an inflammatory condition of the meninges caused by diverse pathogens, remains one of the most severe infections affecting the central nervous system (1). Bacterial meningitis has atypical clinical symptoms, with only 41% of patients exhibiting the typical triad of fever, neck stiffness, and altered mental state (2). Globally, this condition is responsible for approximately 320,000 deaths each year (3), with incidence rates varying dramatically between high-income countries (0.9 cases per 100,000) and resource-limited regions (80 cases per 100,000) (4, 5). In areas with inadequate healthcare systems, mortality rates rise sharply to 54%, and nearly a quarter of survivors experience long-term neurological impairments, such as hearing loss or persistent motor deficits (3–5). This disease continues to pose a significant worldwide health challenge. Consequently, rapid diagnosis and prompt antimicrobial treatment are critical for effective patient management.

Prognosis depends on multiple factors, including microbial virulence, patient age, complications, and the timeliness of appropriate antibiotic therapy (3). The mortality peak for bacterial meningitis occurs within 72 h of onset. Early identification of high-risk patients and implementation of intensive interventions are core strategies to improve prognosis (6). Traditional assessment relies on doctors’ experiential judgment, which is prone to bias due to vague indicator weights. This study aims to identify key predictors of mortality and develop an intuitive prognostic nomogram, providing clinicians with a practical, evidence-based tool for risk assessment and decision-making.

## Materials and methods

### Study design and population

A retrospective cohort study was performed on 277 patients (male: 141 patients) diagnosed with bacterial meningitis at a tertiary care hospital in China from January 2016 to December 2024. The inclusion criteria for the study included (7, 8): microbiological confirmation (cerebrospinal fluid [CSF] culture positive for bacterial pathogens) and neuroimaging requirement (MRI ruling out parenchymal brain involvement). Clinical features required at least one of the following: fever (≥38 °C), signs of intracranial hypertension (headache, vomiting, and altered consciousness), meningeal irritation (nuchal rigidity and Brudzinski’s/Kernig’s signs), CSF findings (leukocytes >10 × 10 (6)/L, protein >0.4 g/L, glucose <2.5 mmol/L or CSF-to-blood glucose ratio <0.6), and age ≥18 years, and regardless of multidrug-resistant (MDR) infection status. The exclusion criteria included: (1) non-bacterial etiologies (viral, fungal, tuberculous, and parasitic meningitis) and (2) missing essential clinical or laboratory data.

### Data collection

Variables extracted from electronic records included: demographics, such as age, sex, and comorbidities (diabetes and immunosuppression). Clinical data included: symptom onset, neurological deficits (e.g., focal weakness), and complications (seizures and ischemic stroke). Laboratory data include CSF: Cell count, biochemistry, Gram stain, culture. Serum: white blood cell (WBC), C-reactive protein (CRP), procalcitonin (PCT), and blood glucose. Neuroimaging: MRI-detected abnormalities (hydrocephalus, empyema).

Patients were categorized based on the origin of infection: symptoms developing within 48 h of hospital admission were classified as community-acquired (CAM), whereas symptoms emerging more than 48 h after admission (8) were classified as hospital-acquired (HAM).

### Statistical analysis

Analyses were performed using SPSS 21.0 and R 4.3.1. Normally distributed continuous data are presented as mean ± SD and compared using t-tests, whereas non-normally distributed continuous data are presented as median [IQR] and compared using Mann–Whitney U-tests. Categorical variables are presented as counts (%) and compared using χ^2^ or Fisher’s exact tests, as appropriate.

The dataset was randomly split into training (70%) and validation (30%) cohorts. Least Absolute Shrinkage and Selection Operator (LASSO) regression was applied to the training cohort to reduce dimensionality and identify the key predictive variables. Selected variables from LASSO were incorporated into a multivariable logistic regression to identify independent factors (Supplementary Figure S1). Model performance was evaluated by AUC, discrimination, and calibration. A two-tailed *p*-value of < 0.05 was considered statistically significant.

## Results

### Baseline characteristics and laboratory findings

The analysis included 277 patients, with 81 fatalities (29.2%). Participants had a median age of 53 years (interquartile range (IQR): 41–62), predominantly male (71.8%, *n* = 199). Random allocation split the cohort into training (70%, *n* = 194) and validation (30%, *n* = 83) cohorts, with no significant intergroup differences (*p* > 0.05).

### Outcome-based comparisons

In the training cohort, 30-day outcomes classified patients into survivors (*n* = 137) and non-survivors (*n* = 57). The latter group exhibited higher rates of intensive care unit (ICU) admission, impaired consciousness, elevated intracranial pressure, neurological sequelae, and MDR infections (all *p* < 0.05). No significant differences were observed in demographics (age and sex), comorbidities (hypertension and diabetes), or clinical features (fever and meningeal signs) (all *p* > 0.05). CSF analysis revealed that elevated WBC, elevated neutrophil proportion, elevated protein, and reduced lymphocyte percentage, glucose in non-survivors (all *p* < 0.05). In addition, serum markers (CRP and PCT) and CSF-to-blood glucose ratios differed significantly (*p* < 0.05), except for peripheral WBC and blood glucose (*p* > 0.05) (Table 1).

**Table 1:** Clinical characteristics and laboratory indicators.

Characteristic	Survivor *n* = 137	Non-survivor *n* = 57	p-value
Age	52 (41, 62)	57 (41, 63)	0.104
Gender	98 (71.5%)	43 (75.4%)	0.578
ICU admission	92 (67.2%)	49 (86.0%)	0.007
Nosocomial infection	125 (91.2%)	52 (91.2%)	0.999
Central infection symptoms	112 (81.8%)	52 (91.2%)	0.096
Consciousness disorder	102 (74.5%)	51 (89.5%)	0.020
Hypertension	76 (55.5%)	32 (56.1%)	0.932
Diabetes mellitus	35 (25.5%)	9 (15.8%)	0.139
Brain tumor	31 (22.6%)	14 (24.6%)	0.771
Cerebral infarction	20 (14.6%)	13 (22.8%)	0.166
Cerebral hemorrhage	81 (59.1%)	42 (73.7%)	0.055
Other Comorbidities	95 (69.3%)	43 (75.4%)	0.393
Fever	117 (85.4%)	51 (89.5%)	0.448
Symptoms of intracranial hypertension	113 (82.5%)	55 (96.5%)	0.009
Meningeal irritation sign	52 (38.0%)	26 (45.6%)	0.322
Invasive cranial procedure	19 (13.9%)	11 (19.3%)	0.341
Neurological complications	33 (24.1%)	28 (49.1%)	<0.001
Craniocerebral trauma	21 (15.3%)	7 (12.3%)	0.582
Multidrug-resistant bacterial infection	44 (32.1%)	39 (68.4%)	<0.001
WBC (CSF)	285 (77, 1906)	5,356 (890, 21,988)	<0.001
N (CSF)	63 (49, 83)	85 (78, 92)	<0.001
L (CSF)	21 (6, 37)	4 (1, 14)	<0.001
Glucose (CSF)	2.25 (1.11, 3.54)	1.11 (1.11, 2.09)	<0.001
Protein (CSF)	2.94 (1.10, 3.00)	3.00 (3.00, 3.00)	<0.001
CL (CSF)	118 (112, 123)	115 (108, 121)	0.077
WBC (blood)	10.4 (7.1, 14.4)	11.6 (8.3, 14.5)	0.256
Glucose (blood)	6.5 (5.3, 8.8)	7.2 (6.0, 8.6)	0.217
Glucose ratio (CSF/blood)	0.37 (0.21, 0.50)	0.20 (0.15, 0.29)	<0.001
CRP (blood)	53 (31, 86)	86 (62, 105)	<0.001
PCT (blood)	0.17 (0.08, 0.46)	0.59 (0.16, 2.14)	<0.001

CSF, cerebrospinal fluid; WBC, white blood cell; N, neutrophil; L, lymphocyte; CRP, C-reactive protein; PCT, procalcitonin.

### Predictors of mortality

**Figure 1:**
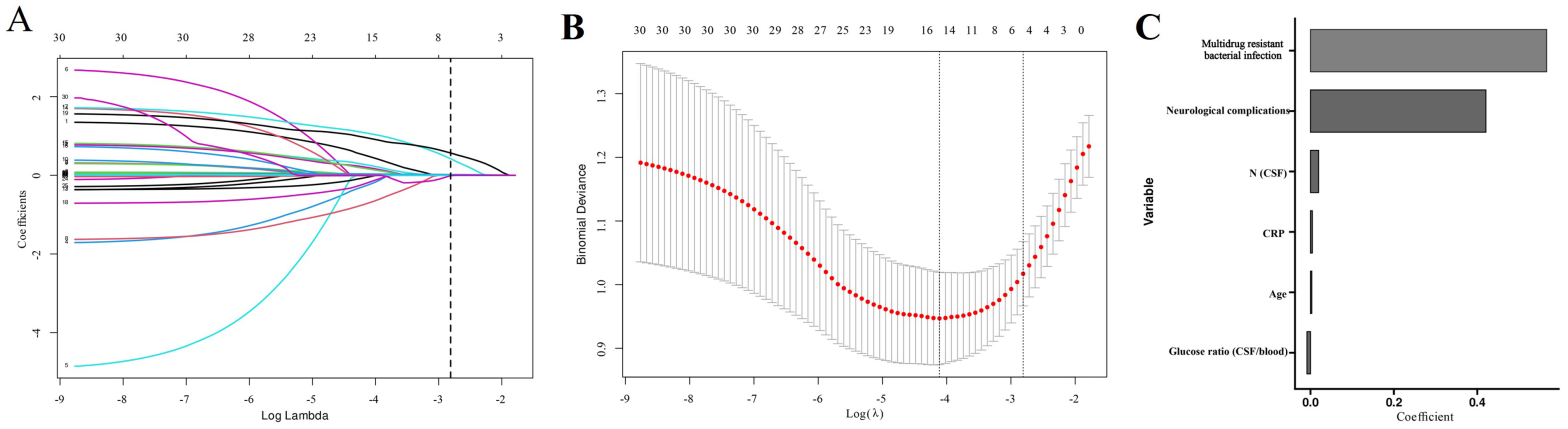
LASSO regression analysis for identifying risk factors. **(A)** The variability of coefficients with variable parameters, **(B)** LASSO regression cross-validation curve, and **(C)** key factors in bacterial meningitis.

**Figure 2:**
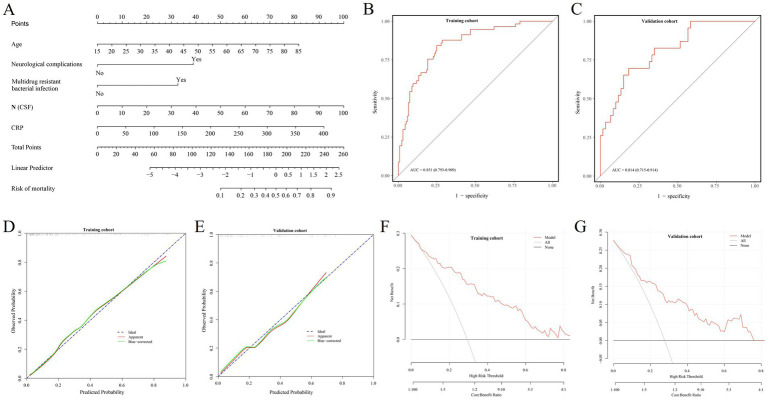
Performance of the nomogram. **(A)** Nomogram, **(B)** ROC curve in the training cohort, **(C)** ROC curve in the validation cohort, **(D)** calibration curve in the training cohort, **(E)** calibration curve in the validation cohort, **(F)** DCA curve in the training cohort, and **(G)** DCA curve in the validation cohort.

From 30 candidate variables, LASSO regression selected six important predictors of 30-day mortality: age, MDR infections, neurological complications, CSF neutrophils, CRP, and CSF-to-blood glucose ratio (Figure 1). Multivariable regression confirmed that age (hazard ratio [HR]: 1.045 [1.014–1.077]), MDR infections (HR: 3.412 [1.508–7.722]), neurological complications (HR: 4.311 [1.871–9.930]), CSF neutrophil (HR: 1.038 [1.016–1.061]), CRP (HR: 1.009 [1.002–1.016]) as significant (all *p* < 0.05; Table 2). Finally, based on the results of the multivariate regression analysis, we established a nomogram for the model (Figure 2A). A nomogram incorporating these predictors achieved an AUC of 0.851 (95% CI: 0.793–0.909). Calibration curves and decision curve analysis (DCA) showed strong agreement between predicted and observed outcomes, as well as clinical utility (Figures 2B,D,F). AUC (0.814, 95% CI: 0.715–0.914), calibration curves, and DCA were further validated to assess the model’s clinical utility in the validation cohort (Figures 2C,E,G).

**Table 2:** Multivariate analysis of risk factors for mortality in model groups.

Variables	HR	95% CI	*p*-value
Age	1.045	1.014–1.077	0.004
Neurological complications	4.311	1.871–9.930	0.001
Multidrug-resistant bacterial infection	3.412	1.508–7.722	0.003
CSF N	1.038	1.016–1.061	0.001
CRP	1.009	1.002–1.016	0.016
AUC	0.851		

CSF, cerebrospinal fluid; N, neutrophil; CRP, C-reactive protein; AUC, area under the curve.

### Risk stratification

The model-defined high-risk subgroup had markedly increased 30-day mortality versus low-risk patients (*p* < 0.05).

## Discussion

Previous studies have shown that, although composite vaccination programs worldwide have reduced the incidence of bacterial meningitis, overall patient prognosis has not significantly improved (9–11). In this retrospective study, we identified age, neurological complications, multidrug-resistant bacterial infection, CSF neutrophils (CSF N), and CRP as independent risk factors for 30-day mortality in affected patients. Based on these multivariate findings, we developed a predictive model for 30-day mortality in adult bacterial meningitis patients. The model demonstrated strong predictive performance, allowing clinicians to stratify patients into high- and low-risk groups and to implement timely interventions for those at high risk, potentially improving outcomes.

The study by McGill et al. (6) reported a mortality rate of up to 30% for adult bacterial meningitis in recent decades, consistent with our findings. Age was a significant prognostic factor, as the deceased group had a higher mean age (*p* = 0.004), and it also emerged as an independent risk factor, aligning with prior studies (12, 13).

Bacterial meningitis typically presents with elevated cerebrospinal fluid (CSF) leukocytes, decreased CSF glucose, and elevated CSF protein (8). Our study revealed that deceased patients had higher WBC, higher protein levels, and lower CSF glucose. However, in hyperglycemic patients, elevated blood glucose may artificially normalize CSF glucose levels, making the CSF-to-blood glucose ratio a more reliable indicator. Our results showed no significant difference in blood glucose between groups but a marked difference in the CSF-to-blood glucose ratio, corroborating the findings of Hegen et al. (14).

CRP is a marker of inflammation and immunity. Waterfiend et al. (15) demonstrated its diagnostic accuracy in predicting bacterial meningitis mortality, consistent with our observation of statistically significant differences in CRP levels between survivors and non-survivors (*p* < 0.05).

Neurological complications—such as cerebral infarction, brain abscess, hydrocephalus, and focal deficits (e.g., hearing loss, cognitive impairment, and seizures)—are common in bacterial meningitis (16). Our study revealed a significantly higher incidence of neurological complications in the deceased group (*p* < 0.001), supporting Edmond et al. (17) and Ramakrishnan et al. (18), who linked these complications to higher mortality and neuropsychological sequelae.

All patients in this study were confirmed by CSF culture positivity. Gram-negative bacteria accounted for 71.84% (*n* = 199), while Gram-positive bacteria comprised 28.16% (*n* = 78). The top three Gram-negative isolates were Acinetobacter baumannii, Klebsiella pneumoniae, and Pseudomonas aeruginosa. Antimicrobial susceptibility testing showed limited resistance among Gram-positive bacteria, with no vancomycin-resistant staphylococci or enterococci detected. In contrast, Gram-negative isolates exhibited widespread resistance, including 119 (42.96%) multidrug-resistant infections, predominantly *Acinetobacter, Pseudomonas*, and *Enterobacterales*. Multidrug-resistant infection bacterial meningitis poses significant therapeutic challenges, with A. baumannii meningitis mortality rates reaching 15–70%, underscoring its prognostic impact (19).

This single-center retrospective study may carry selection bias. Retrospective design also precluded the inclusion of newer biomarkers (e.g., IL-6), potentially affecting model performance. The small sample size and lack of external validation may limit generalizability, warranting future multicenter prospective studies for refinement.

In conclusion, by integrating clinical features, laboratory data, and microbiological findings, we used LASSO regression to identify five independent predictors of 30-day mortality in bacterial meningitis and developed a novel predictive model with strong performance. This tool may facilitate early risk stratification and guide timely interventions to improve patient outcomes.

## Data Availability

The raw data supporting the conclusions of this article will be made available by the authors, without undue reservation.
