# Supplementary figures and images for: Development and validation of a predictive model for 30-day mortality in adult bacterial meningitis: a retrospective cohort study
Source: Front Med (Lausanne). 2025 Nov 20;12:1692631. doi: 10.3389/fmed.2025.1692631 (PMC12675203; doi:10.3389/fmed.2025.1692631)

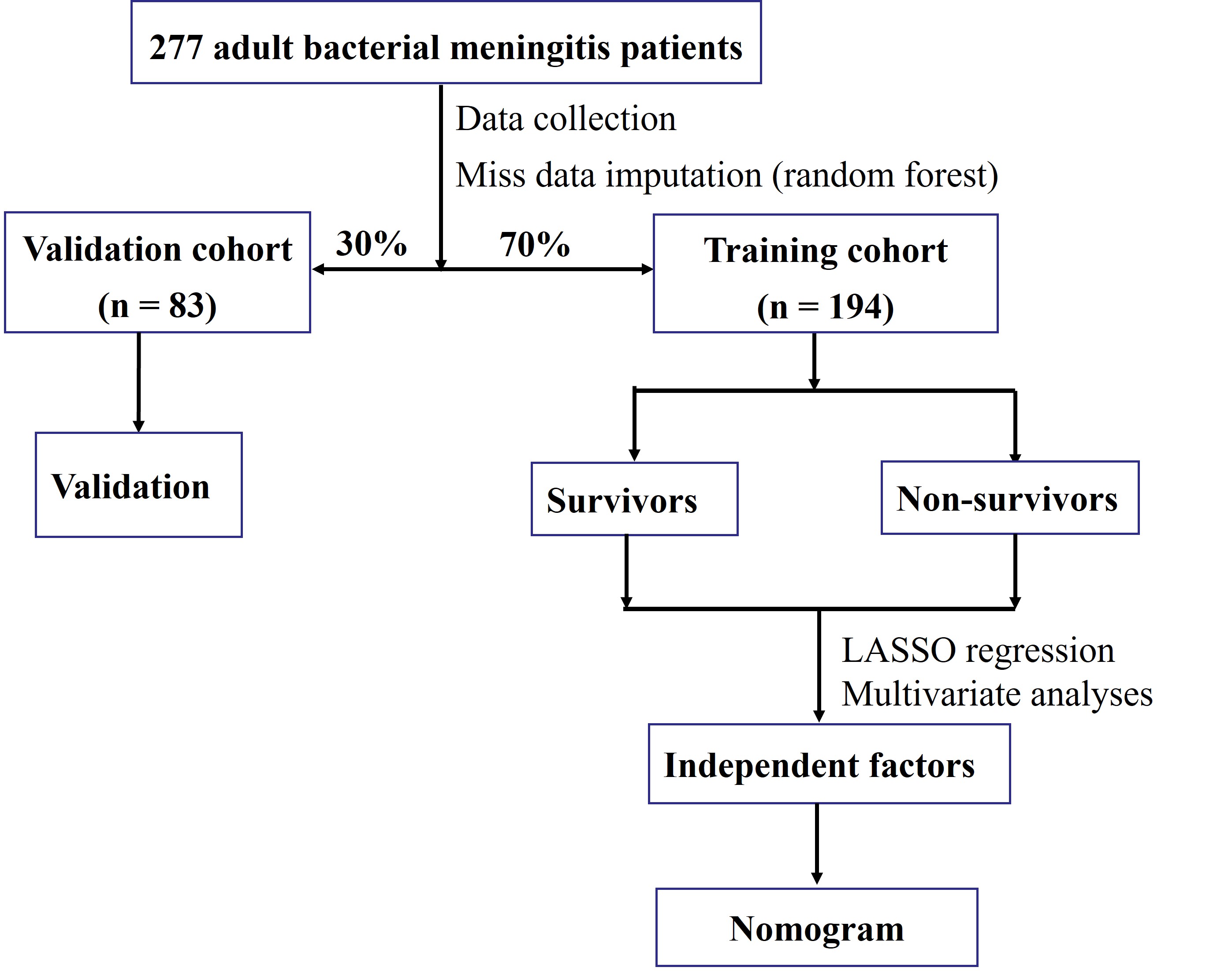

Supplement: Supplementary file 1 [file Image_1.JPEG]
